# Daily Fluctuations in Smartphone Use, Psychological Detachment, and Work Engagement: The Role of Workplace Telepressure

**DOI:** 10.3389/fpsyg.2018.01808

**Published:** 2018-09-24

**Authors:** Michelle Van Laethem, Annelies E. M. van Vianen, Daantje Derks

**Affiliations:** ^1^Department of Work and Organizational Psychology, University of Amsterdam, Amsterdam, Netherlands; ^2^Department of Work & Organizational Psychology, Erasmus University Rotterdam, Rotterdam, Netherlands

**Keywords:** workplace telepressure, smartphone use, psychological detachment, work engagement, day-level relations

## Abstract

Today’s work environment is shaped by the electronic age. Smartphones are important tools that allow employees to work anywhere and anytime. The aim of this diary study was to examine daily smartphone use after and during work and their association with psychological detachment (in the home domain) and work engagement (in the work domain), respectively. We explored whether workplace telepressure, which is a strong urge to respond to work-related messages and a preoccupation with quick response times, promotes smartphone use. Furthermore, we hypothesized that employees experiencing high workplace telepressure would have more trouble letting go of the workday during the evening and feel less engaged during their workday to the extent that they use their smartphone more intensively across domains. A total of 116 employees using their smartphones for work-related purposes completed diary questionnaires on five workdays (*N* = 476 data points) assessing their work-related smartphone use, psychological detachment after work, and engagement during work. Workplace telepressure was measured as a between-individual variable and only assessed at the beginning of the study, as well as relevant control variables such as participants’ workload and segmentation preference (a preference for work and home domains to be as segmented as possible). Multilevel path analyses revealed that work-related smartphone use after work was negatively related to psychological detachment irrespective of employees’ experienced workplace telepressure, and daily smartphone use during work was unrelated to work engagement. Supporting our hypothesis, employees who reported high telepressure experienced less work engagement on days that they used their smartphone more intensively during work. Altogether, intensive smartphone use after work hampers employees’ psychological detachment, whereas intensive smartphone use during work undermines their work engagement only when employees experience high workplace telepressure as well. Theoretical and practical implications of these findings are discussed.

## Introduction

Communication technology has an ubiquitous role in our daily working lives: many employees cannot perform their job without using computer facilities. Smartphones serve as small computers and include a variety of functions such as phone calls, digital calendars, internet and social media access, and most importantly sending and receiving e-mails (Middleton, 2007). For example, 97% of Dutch employees have a smartphone with internet access and 94% use their smartphone daily for internet activities (CBS, 2018). In the past 5 years, internet access via smartphones has increased by approximately 25%.

Communication technology devices have enabled employees to bring work tasks into the home domain thereby facilitating work flexibility, with the blurring of boundaries between work and home domains as a side effect (Demerouti et al., 2014). The following scenario may sound very familiar to many of us: imagine sitting in your living room in the evening watching television and relaxing after a busy day at work when you suddenly receive an urgent e-mail from your boss on your smartphone. What will you do: answer the e-mail right away or wait until the next day? Some of us would respond to the urgent e-mail, whereas others would not or would not have seen the e-mail anyway.

Employees who feel a strong urge to respond to work-related messages while wanting to respond quickly (i.e., the ones who would respond to their boss in the described scenario) experience high workplace telepressure (Barber and Santuzzi, 2015). Workplace telepressure is a relatively new concept in a rapidly developing modern working world and a timely topic to study. It is thus essential to develop theory on how workplace telepressure may impact employees’ involvement in work activities after and during work.

In this study, we first expect that workplace telepressure will motivate employees to use their smartphone on a daily basis for work-related purposes during off-job hours. However, high work involvement in the form of spending time on work activities while being at home could have its price for individuals. That is, work-related smartphone use after work may impede employees to detach and recover from their work during the following evening, which could ultimately harm their (mental) health (Derks et al., 2014). It is possible, however, that employees have particularly difficulties with psychologically detaching from their work (i.e., mentally switching off work) when they feel pressured to stay ‘online,’ thus when they experience high workplace telepressure. Besides using their smartphone more frequently, these employees stay mentally occupied because of their constant alertness to incoming messages. In this study, we propose that high workplace telepressure will strengthen the negative relationship between work-related smartphone use and psychological detachment at home.

Additionally, we expect that workplace telepressure will motivate employees to use their smartphone frequently when being at work (Barber and Santuzzi, 2015; Grawitch et al., 2017). However, frequent smartphone use at work may undermine high involvement at work, referred to as work engagement (Bakker et al., 2008), particularly if employees feel pressured to use their smartphone and thus may view their frequent smartphone use as a burden. In contrast, if employees feel no such pressure and rather perceive their smartphone as a resource that increases their autonomy, flexibility and functioning, frequent smartphone use at work may relate to higher work engagement.

**FIGURE 1:**
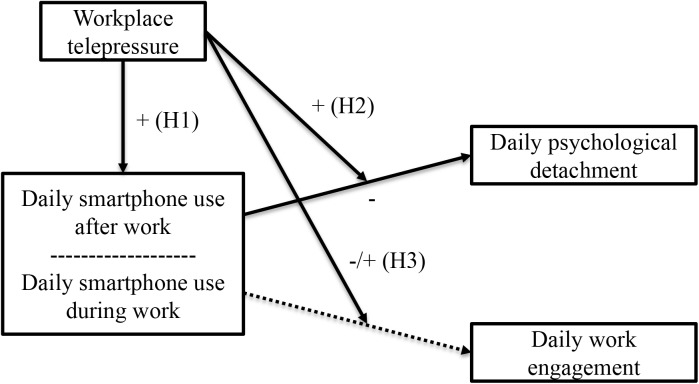
Visualization of the research model.

In this study we propose that the relationship between smartphone use and work engagement will be negative when experienced workplace telepressure is high whereas this relationship will be positive when experienced workplace telepressure is low. See **Figure 1** for a visualization of the research model.

Although workplace telepressure and the use of smartphones for work-related purposes have become important topics for employees and employers alike, research on these topics is yet relatively sparse (Richardson, 2017). This study aims to fill this void and consequently contributes to theory and practice. Moreover, the few prior studies on work-related smartphone use have mainly focused on work-related smartphone use during off-job hours (Schlachter et al., 2017). The present study aims to add to this line of research by simultaneously examining daily work-related smartphone use during off-job hours and during work hours. Employees who feel pressured to frequently use their smartphone at work may experience less control over their work, which may undermine rather than promote their work engagement (MacCormick et al., 2012). This is the first study that test this proposition.

Investigating the correlates of workplace telepressure and frequent smartphone use off and during work is important for developing theory on human adaptation to technological devices. In addition, this investigation is extremely relevant for society and organizations, because more knowledge is needed about the possible detrimental or beneficial outcomes of employees’ cognitions pertaining to the use of smartphones for work-related activities and employees’ actual use of these devices. This knowledge may help to develop evidence-based interventions of how employees can deal with devices so that these devices do not cause them stress but rather help them to stay engaged.

Because work-related smartphone use, psychological detachment, and work engagement are prone to fluctuate from day to day, we test our propositions using a daily diary design, allowing us to examine relationships across multiple time points (i.e., during several days), and capture “life as it is lived” (Bolger et al., 2003, p. 597). A diary approach allows us to closely follow an employee’s behavior during work and off-job time and is essential in studying short-term processes like work-related smartphone use (Ohly et al., 2010).

### Workplace Telepressure and Work-Related Smartphone Use

We have already established that almost every employee in Western societies has a smartphone with access to (work) e-mail (CBS, 2018; Statista, 2018). Many of them use their smartphone for work purposes while being off work. To be precise, 44% of American employees admit to checking their work e-mail during vacation and 54% do so while being sick at home (American Psychological Association, 2013). Apparently, these employees are in one way or another motivated to use their smartphone for work in their private time.

Barber and Santuzzi (2015) coined the term workplace telepressure and defined it as a preoccupation with and urge to respond promptly to work-related messages. These authors further showed that workplace telepressure was primarily a function of external pressures that employees experience, such as prescriptive norms in the organization. Other researchers (Grawitch et al., 2017), however, found that also personal pressures, such as neuroticism, workaholism, and low self-control, contributed to the experience of workplace telepressure, all personality variables that are conceived of as detrimental for employees’ well-being and health.

Employees who experience workplace telepressure, whether this is from external or internal pressures, might be more likely to give in to their urges and use their smartphone for work-related purposes in their spare time more often than employees who do not experience this pressure. Experienced pressures are difficult to resist, even if one realizes that the behavioral response to the pressure is unnecessary or could be harmful (Baumeister et al., 2007). Indeed, prior research found that employees who experienced high workplace telepressure also reported increased e-mail responding (Barber and Santuzzi, 2015; Grawitch et al., 2017).

Extant research on workplace telepressure and work-related smartphone use mostly focused on work-related smartphone use *during off-job time* (see Schlachter et al., 2017, for a review). As smartphones have become a constant companion in our daily working lives it is also important to examine work-related smartphone use *during work hours* and how this relates to beneficial work outcomes. Many employees own a smartphone that they use during work hours. For instance, a recent survey by Steelcase showed that 47% of Dutch employees are equipped with mobile phones by their employers (Steelcase, 2016). Based on prior research showing a positive relationship between workplace telepressure and work-related smartphone use *after* work (Barber and Santuzzi, 2015), it is plausible to expect a relationship between workplace telepressure and work-related smartphone use *during* work. Since we examine smartphone use at the day-level (rather than in general), we propose:

***Hypothesis 1:*** *Workplace telepressure is positively related to day-level work-related smartphone use after work (a) and during work (b).*

### Work-Related Smartphone Use After Work and Psychological Detachment

Previous studies on work-related smartphone use during off-job time have examined and found support for psychological detachment as a direct consequence of work-related smartphone use and as a mechanism linking smartphone use to recovery and well-being as well as work-family conflict (Park et al., 2011; Derks et al., 2012, 2014; Barber and Jenkins, 2014; Lanaj et al., 2014; Ohly and Latour, 2014; Schlachter et al., 2017). Psychological detachment refers to being able to mentally disengage from work during off-job time (Sonnentag and Fritz, 2007). Employees who psychologically detach from work do not think of their work during free time. According to Effort-Recovery theory (Meijman and Mulder, 1998) and Allostatic Load theory (McEwen, 1998), recovery during non-work hours is crucial for allowing stress-related psycho-physiological reactions to return to baseline or pre-demand levels. Thus, being preoccupied with responding to work-related messages when at home, on vacation, or while being sick may hamper psychological unwinding, which may have negative consequences for recovery and well-being, and ultimately work performance.

Although research has shown that employees tend to have more trouble detaching form work when they use their smartphone for work during off-job hours (Derks et al., 2014), it is possible that especially employees who experience high workplace telepressure are unable to mentally switch of work when intensively using their smartphone after work. These employees are not only actually involved in work activities when using their smartphone but they are also constantly alert to receiving and responding to other work-related emails that might arrive. In addition, they may perceive lower vigilance and non-response to emails (i.e., detachment) as failure or improper work behavior. Their frequent smartphone use in combination with a state of mental alertness keep them attached to work (e.g., Walsh et al., 2010; Vorderer et al., 2016; Sonnentag, 2017). Conversely, employees who use their smartphone (occasionally) frequently but without experiencing workplace telepressure will detach from this work-related activity as soon as they have finished using their smartphone. These employees are less preoccupied with the possible arrival of work-related messages than their pressured counterparts and are thus able to switch off their work as soon as they can. We hypothesize the following:

***Hypothesis 2:*** *Workplace telepressure moderates the negative relationship between daily work-related smartphone use during off-job hours and daily psychological detachment in such a way that experiencing high workplace telepressure strengthens the negative relationship (a) and experiencing low workplace telepressure weakens the negative relationship (b).*

### Work-Related Smartphone Use During Work and Work Engagement

Above, we argued that workplace telepressure not only promotes work-related smartphone use after work but also smartphone use at work. Here we discuss how smartphone use at work may relate to employees’ work engagement and whether this relationship depends on employees’ experienced workplace telepressure. Work engagement is “a positive, fulfilling, affective-motivational state of work-related well-being that is characterized by vigor, dedication, and absorption” (Bakker et al., 2008, p. 187). Employee work engagement is regarded vital for both employees and organizations (Bakker et al., 2008; Gruman and Saks, 2011). Employees who are strongly engaged with their work feel dedicated and energetic and are intrinsically motivated to develop themselves and to perform to the best of their abilities, which in turn, make them an asset for the productivity and performance of the organization. In addition, although employee work engagement has been studied as a trait characteristic, diary studies have shown that it can fluctuate over time as caused by day-level job demands and resources (e.g., Xanthopoulou et al., 2009; Sonnentag et al., 2010; Breevaart et al., 2012).

Building on the Job Demands-Resources (JD-R) model (e.g., Bakker et al., 2014), we expect that the combination of daily work-related smartphone use and workplace telepressure will relate to daily work engagement. The JD-R model categorizes job characteristics as job demands or job resources, with job demands being relatively stronger predictors of burnout (the health impairment process) and job resources being relatively stronger predictors of work engagement (the motivational process) (Llorens et al., 2006; Schaufeli and Taris, 2014).

Smartphone use at work can be either seen as a job demand or a job resource (Day et al., 2010). Employees may experience access to a smartphone as a job demand when they associate it with increased workload and longer working hours (Peters et al., 2009; Demerouti et al., 2014). Although job demands tend to be relatively less strongly associated with work engagement, frequent smartphone use may nonetheless hamper employee work engagement when perceived as a demand. Conversely, employees may experience access to a smartphone as a job resource when they associate it with flexibility and increased autonomy over work tasks (Day et al., 2010). If this is the case, frequent work-related smartphone use may relate to increased work engaement.

Whether employees experience smartphone use at work as a job demand or job resource may depend on the extent to which they experience workplace telepressure. Employees who experience high workplace telepressure may perceive their smartphone as a hindering job demand, whereas employees who do not experience this pressure may perceive their smartphone as a helpful device (i.e., a job resource). As outlined above, high workplace telepressure can stem from external pressures (e.g., prescriptive norms) or disadvantageous internal pressures (neuroticism, workaholism, low self-control). These pressures reflect job and personal demands, respectively, rather than resources and will thus negatively impact the relationship between employee smartphone use and work engagement. Intensive smartphone use combined with high workplace telepressure will reduce work engagement, whereas intensive smartphone use combined with low workplace telepressure will increase it. We hypothesize:

***Hypothesis 3****: Daily work-related smartphone use during work is negatively related to daily work engagement for individuals experiencing high workplace telepressure (a) and positively related to day-level work engagement for individuals experiencing low workplace telepressure (b).*

## Materials and Methods

### Procedure and Participants

Participants were recruited via e-mail, phone, LinkedIn, or other social media outlets, resulting in a heterogeneous convenience sample of Dutch employees. To be included in the study, potential participants had to work at least 3 days a week within the same organization and had to use their smartphone for work-related purposes. In-depth information about the data collection as well as anonymity and confidentiality of responses was provided in an e-mail and the informed consent at the beginning of the study. This study was carried out in accordance with the guidelines formulated by the Ethics Review Board of the Faculty of Social and Behavioral Sciences, University of Amsterdam, and has been approved by the aforementioned Ethics Review Board (reference number 2017-WOP-8035). All subjects gave written informed consent in accordance with the Declaration of Helsinki. Participation was entirely voluntary and could be stopped at any time. Participants had the chance to win one of four gift certificates each worth €25. Their chance of winning was dependent on the amount of completed questionnaires. This approach was chosen to minimize participant dropout.

All data were collected through online questionnaires. Participants provided their responses by using a computer, tablet or smartphone. At the beginning of the study, participants filled out a general questionnaire measuring between-individual variables such as workplace telepressure, segmentation preference, workload, and demographics. Next, participants received five short daily questionnaires on the days they had specified as working days. As some participants worked part-time, working days did not have to be consecutive. In these short questionnaires, day-level (state) variables were assessed (i.e., smartphone use during and after work, work engagement during work, psychological detachment after work). Daily questionnaires were always sent in the evening at 20:00. If participants had not responded until 22:30 they received a reminder. The link to the questionnaire expired the following morning at 04:00 to prevent participants from responding during the next working day. In the case that participants had filled out less than three daily questionnaires, they were invited twice more to fill out an additional questionnaire.

Of the 192 employees who completed the general questionnaire, 116 participated in the daily diary study and filled out at least three daily questionnaires. Of the 580 distributed daily questionnaires (116 participants × 5 days) 82% were completed, resulting in 476 data points at the within-person level. Mean age of participants (55.2% females) was 38.1 (*SD* = 12.7). Most participants had received higher professional or university education (88.8%) and worked fulltime (56.9%). Professional backgrounds were diverse, such as teaching (22.4%), healthcare (15.5%), and ICT (11.2%). All participants indicated to use their smartphone for work-related purposes during work and had access to their work after working hours.

### Measures

#### Between-Individual Measures

The between-individual measures at the start of the study were: workplace telepressure, segmentation preference, workload, and demographics.

*Workplace telepressure* was measured with the six-item scale developed by Barber and Santuzzi (2015). The items were preceded by an introductory statement: “When responding to the following statements, think about how you use technology to communicate with people in your workplace. Specifically think about message-based technologies that allow you to control when you respond (email, text messages, voicemail, etc.). Please rate how much you agree or disagree with the statements. When using message-based technology for work purposes …” A sample item is “I can concentrate better on other tasks once I’ve responded to my messages”. All items were rated on a five-point scale ranging from 1 (*strongly disagree*) to 5 (*strongly agree*). Cronbach’s alpha for the scale was 0.92.

#### Control Variables

*Workload* is a variable that potentially may act as a confounding variable when examining work-related smartphone use and was thus included in this research. Workload was measured with a three-item scale (Bakker et al., 2003). A sample item is “I have to work extra hard to finish things.” All items were rated on a five-point scale ranging from 1 (*never*) to 5 (*very often*). Cronbach’s alpha for the scale was 0.87.

Employees may also vastly differ in how they prefer to handle their work and home domains (Park et al., 2011; Derks et al., 2016). Therefore, *segmentation preference* may potentially act as a confounding variable when examining work-related smartphone use and detachment. Segmentation preference was assessed with the four-item subscale segmentation preference from Kreiner (2006). An example item is “I like to be able to leave work behind when I go home” and the response categories ranged from 1 (*totally disagree*) to 5 (*totally agree*). A higher score indicated a preference for keeping work and home domains separate. Cronbach’s alpha was 0.82.

*Demographics* included gender, age, and educational level.

#### Within-Individual Measures

The within-individual measures included in the five daily questionnaires were: smartphone use during work, smartphone use after work, work engagement during work, and psychological detachment after work. Factor analyses confirmed that all scales measured different constructs.

*Smartphone use during work* was assessed with the smartphone use scale from Derks and Bakker (2014). The four items were adjusted for daily measurement and referred to smartphone use during work. The items were preceded by a short introductory statement: “The following statements concern your smartphone use for work-related purposes during working hours.” A sample item is “Today, I used my smartphone intensively during work hours.” All items were rated on a five-point scale ranging from 1 (*totally disagree*) to 5 (*totally agree*). Cronbach’s alpha coefficients varied from 0.74 to 0.86 with an average of 0.82 across the different days.

*Smartphone use after work* was assessed in a similar way as smartphone use during work. The same four-item scale from Derks and Bakker (2014) was used, which now referred to smartphone use after work. Cronbach’s alpha coefficients varied from 0.79 to 0.86 with an average of 0.83 across the different days.

*Work engagement during work* was measured with the nine-item State Work Engagement Scale (Breevaart et al., 2012). An example item is “Today, my job inspired me” and responses were provided on a seven-point scale ranging from 0 (*strongly disagree*) to 6 (*strongly agree*). Cronbach’s alpha coefficients ranged from 0.89 to 0.93 with an average of 0.91 across all measurement days.

*Psychological detachment after work* was measured with the four-item psychological detachment subscale of the Recovery Experiences Questionnaire (Sonnentag and Fritz, 2007), which was adjusted for daily measurement. An example item is “In my free time after work I forgot about work today” and the response categories ranged from 1 (*totally disagree*) to 5 (*totally agree*). Cronbach’s alpha coefficients ranged from 0.81 to 0.92 with an average of 0.88 across all measurement days.

### Statistical Analyses

Repeated daily measurements were nested within individuals. Intra-class correlations indicated that 36% of the variance in work-related smartphone use during work and 31% of the variance in smartphone use after work was on the day-level (within-individual). Moreover, 57% of the variance in work engagement and 53% of the variance in psychological detachment could be attributed to day-level variations. Thus, we concluded that using a multilevel approach was justified.

**FIGURE 2:**
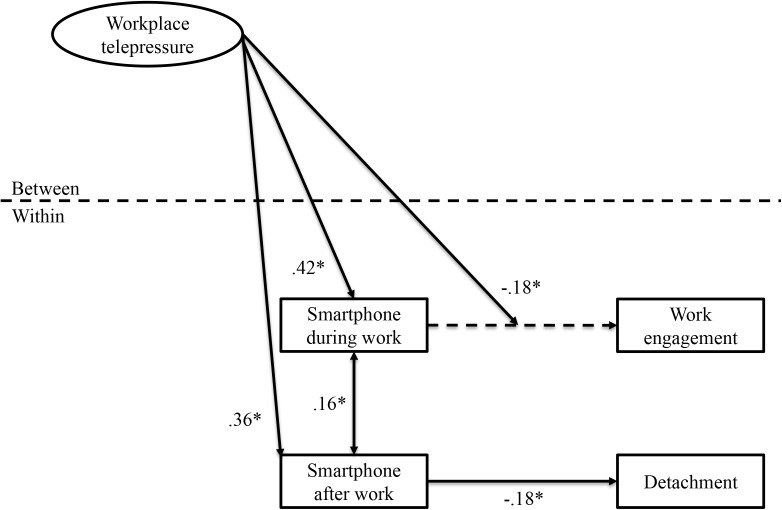
Multilevel model including all significant results. For clarity, insignificant pathways and control variables are not depicted. ^∗^*p* < 0.05.

Day-level variables were modeled as level-1 variables (*N* = 476 daily measurements) and individuals as level-2 variables (*N* = 116 participants). Following the recommendations by Aguinis et al. (2013), we grand mean centered workplace telepressure because this variable was the only predictor in our model (see **Figure 2**) and modeled at the between-level. We also grand-mean centered our control variables age, workload and segmentation preference. We performed multilevel path analysis with ML estimation in Mplus 7.4 (Muthén and Muthén, 2015) to examine day-level relations between work-related smartphone use during and after work, work engagement, and psychological detachment, and workplace telepressure as cross-level predictor. All variables were entered in the same model: work-related smartphone use during and after work, work engagement, and psychological detachment were modeled at the within level. Workplace telepressure and control variables were modeled at the between level.

## Results

**Table 1** presents descriptive statistics and between-level correlations among the study variables. Work-related smartphone use during work was positively related to work-related smartphone use after work (*r* = 0.40, *p* < 0.01). When participants used their smartphone for work-related purposes during work, they also engaged more often in work-related smartphone use after work.

**Table 1:** Means, standard deviations, and intercorrelations among all study variables.

	*M*	*SD*	ICC	1	2	3	4	5	6	7	8	9
(1) Gender (% male)	44.8%											
(2) Age	38.14	12.71		0.08								
(3) Educational level^a^ (1–5)	4.34	0.75		0.13	-0.07							
(4) Workload (1–5)	3.95	0.85		0.12	0.13	0.05						
(5) Segmentation preference (1–5)	3.21	0.83		0.07	-0.21^∗^	-0.06	-0.17					
(6) Workplace telepressure (1–5)	2.95	0.86		0.10	-0.18	0.18^∗^	-0.01	0.22^∗^				
(7) Smartphone use after work (1–5)	2.72	0.86	0.69	0.09	0.03	0.03	0.16	-0.12	0.30^∗∗^			
(8) Smartphone use during work (1–5)	2.90	0.82	0.64	-0.09	0.07	0.18	0.16	-0.18^∗^	0.33^∗∗^	0.40^∗∗^		
(9) Psychological detachment (1–5)	3.30	0.72	0.47	-0.01	-0.07	-0.07	-0.40^∗∗^	0.27^∗∗^	-0.05	-0.26^∗∗^	-0.15	
(10) Work engagement (0–6)	4.73	0.79	0.43	-0.08	0.10	-0.09	-0.09	-0.38^∗∗^	-0.15	-0.01	0.12	0.10


*^∗^p < 0.05, ^∗∗^p < 0.01. ^*a*^categories: 1 = primary education, 2 = high school education, 3 = secondary vocational education, 4 = higher professional education, 5 = university education N = 116 individuals, N = 476 data points. Correlations between day-level variables are average scores across all five measurement days. M, mean; SD, standard deviation; ICC, intra-class correlation. Means, standard deviations, and correlations were calculated with the statistical program JASP (JASP Team, 2018)*.

Demographic variables were unrelated to smartphone use, psychological detachment, and work engagement. The control variable workload was significantly negatively related to psychological detachment (*r* = -0.40, *p* < 0.01), meaning that participants who experienced higher workload reported lower psychological detachment than participants who experienced a relatively lower workload. Segmentation preference was positively related to workplace telepressure (*r* = 0.22, *p* < 0.05) and psychological detachment (*r* = 0.27, *p* < 0.01), but negatively to work-related smartphone use during work (*r* = -0.18, *p* < 0.05) and work engagement (*r* = -0.38, *p* < 0.01). Participants who had a preference for keeping the work and home domains as separate as possible reported higher levels of workplace telepressure, but were able to better mentally detach from work during free time. In addition, participants who had a preference for keeping the work and home domains separate used their smartphones less often for work-related purposes during work and were less engaged during work. Multilevel path analysis confirmed these relations and showed that workload and segmentation preference were potential confounders. Thus, in the further analyses we controlled for workload and segmentation preference as level-2 variables.

### Hypothesis Testing

Following recommendations of Hu and Bentler (1999), the multilevel model including all hypothesized paths fitted the data well, χ^2^ = 2.88, *df* = 2, RMSEA = 0.03, CFI = 0.99, SRMR_within_ = 0.03, SRMR_between_ = 0.01. A second model only including significant hypothesized paths did not fit the data better than the initial model (χ^2^ = 6.72, *df* = 6, RMSEA = 0.02, CFI = 0.99, SRMR_within_ = 0.04, SRMR_between_ = 0.03). Therefore and for completeness, the initial model was kept. Hereafter, the results of this multilevel model are presented (see **Figure 2**).

Supporting hypothesis 1, workplace telepressure related positively to work-related smartphone use during work (γ = 0.42, *SE* = 0.09, *p* < 0.001) and work-related smartphone use after work (γ = 0.36, *SE* = 0.09, *p* < 0.001). Participants reporting high workplace telepressure were more likely to engage in work-related smartphone use during and after work than participants reporting lower workplace telepressure. Workplace telepressure was not related to work engagement during work hours (γ = -0.06, *SE* = 0.10, *p* = *ns*) nor psychological detachment from work during free time (γ = -0.12, *SE* = 0.10, *p* = *ns*).

Hypotheses 2a and 2b proposed that the negative relationship between day-level work-related smartphone use after work and day-level psychological detachment would be moderated by workplace telepressure. As expected, daily work-related smartphone use after work was negatively related to psychological detachment from work during free time (γ = -0.18, *SE* = 0.05, *p* < 0.001). However, results did not reveal a moderating effect of workplace telepressure on the relation between work-related smartphone use after work and psychological detachment from work during free time (γ = -0.02, *SE* = 0.08, *p* = *ns*). Hence, hypotheses 2a and 2b had to be rejected. Rather, daily work-related smartphone use after work seems to impede psychological detachment from work during free time anyway, irrespective of an individual’s experienced telepressure.

**FIGURE 3:**
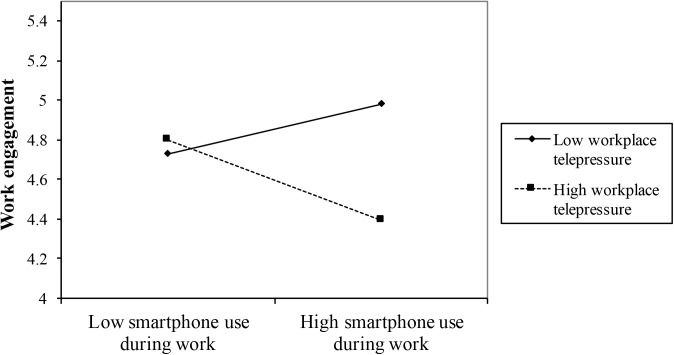
Moderation effect of workplace telepressure (±2 SD) on the relationship between daily work-related smartphone use during work and work engagement.

Hypotheses 3a and 3b proposed that the relationship between day-level work-related smartphone use during work and day-level work engagement would be moderated by workplace telepressure. Daily work-related smartphone use during work was unrelated to experienced work engagement the same day (γ = -0.03, *SE* = 0.05, *p* = *ns*). As proposed, workplace telepressure moderated the relationship between work-related smartphone use during work and work engagement (γ = -0.18, *SE* = 0.09, *p* < 0.05). Simple slopes tests indicated that for participants who experienced high levels of workplace telepressure, work-related smartphone use during work was negatively related to work engagement, but only for participants who reported very high levels of workplace telepressure (+1SD: γ = -0.18, *SE* = 0.11, *p* = *ns*; +2SD: γ = -0.34, *SE* = 0.17, *p* < 0.05). However, for participants who experienced low levels of workplace telepressure, work-related smartphone use during work was unrelated to work engagement (γ = 0.13, *SE* = 0.12, *p* = *ns*). These results largely support hypothesis 3a but do not support hypothesis 3b. See **Figure 3** for a visualization of the moderation effect.

## Discussion

Using a diary approach, we aimed to explore daily smartphone use after and during work and their association with psychological detachment (in the home domain) and work engagement (in the work domain), respectively. Our results contribute to theory and practice by showing that workplace telepressure promotes smartphone use after and during work. In addition, we demonstrated that employees who use their smartphone for work during off-job hours have more trouble letting go of the workday during the evening regardless of whether they experience workplace telepressure. Lastly, we found that employees experiencing high workplace telepressure and use their smartphone intensively during work hours feel less engaged during their workday. These results are largely in line with our hypotheses.

### Workplace Telepressure and Work-Related Smartphone Use

Workplace telepressure predicted work-related smartphone use during off-job hours, but also intensive smartphone use during work. Employees who experienced high workplace telepressure engaged in work-related smartphone use after and during work more often than employees who experienced lower workplace telepressure. Workplace telepressure and work-related smartphone use seem to be closely and positively related as indicated by large effect sizes. These findings are in line with our expectations and support the little research that has been done on workplace telepressure and smartphone use. Employees who experience workplace telepressure, which is driven by both external (e.g., prescriptive norms) and internal pressures (e.g., neuroticism), seem to be more likely to give in to these pressures and the urge to use their smartphones for work. Previous studies have found that high workplace telepressure was related to increased e-mail responding (Barber and Santuzzi, 2015; Grawitch et al., 2017), which fits nicely with our findings as smartphones are predominantly used to send and receive e-mails (Middleton, 2007).

This study extends previous research by showing that workplace telepressure not only impacts work-related smartphone use *during off-job time*, but also *during work hours.* Nowadays, employers frequently equip their employees with smartphones to use during work hours (e.g., to foster flexibility; Steelcase, 2016). Thus, it seems plausible that workplace telepressure also relates strongly to work-related smartphone use during work, which is supported by the findings of this study.

### Work-Related Smartphone Use After Work Hours and Psychological Detachment

Most prior research has focused on psychological detachment as an adverse consequence of work-related smartphone use (e.g., Derks et al., 2012, 2014; Barber and Jenkins, 2014; Schlachter et al., 2017). Corroborating prior findings, daily work-related smartphone use during off-job hours was also negatively related to psychological detachment in our diary study. When employees were using their smartphone intensively for work-related purposes during their free time they were less able to mentally disengage from work. The negative relationship between daily work-related smartphone use after work and psychological detachment also provides support for Effort-Recovery theory (Meijman and Mulder, 1998) and Allostatic Load theory (McEwen, 1998). By frequently using one’s smartphone during non-work hours, psychological unwinding and crucial recovery processes (i.e., allowing stress-related psycho-physiological reactions to return to baseline or pre-demand levels) are hampered. Impeded recovery may deteriorate worker well-being and work performance in the long run (McEwen, 1998; Meijman and Mulder, 1998).

However, contrary to our expectations, the negative relationship between work-related smartphone use and psychological detachment existed irrespective of employees’ experienced workplace telepressure. Experiencing high workplace telepressure did not strengthen the negative relationship, nor did low workplace telepressure weaken the negative relationship. Importantly, this study adds to the body of research on work-related smartphone use during off-job time by showing that smartphone use after work is not favorable for employee detachment and recovery, regardless of workplace telepressure. However, there may be other factors that impact the smartphone-detachment relationship. For example, Derks et al. (2014) have shown that organizational norms regarding separating the work and home domains (i.e., segmentation norm) moderated the relationship between work-related smartphone use and psychological detachment (Derks et al., 2014). Employees reporting a high segmentation norm at work had more trouble to psychologically detach from work when using their smartphone more intensively.

It seems to be especially relevant to further examine what drives an employee’s motivation and alertness to receiving and responding to work-related emails. Some employees can easily switch off their smartphones when at home, while others cannot (see also Derks et al., 2016). Future research may examine whether individual differences (e.g., personality characteristics) and, for example, a conscious handling of devices and greater awareness of health-related risks significantly impact the smartphone-detachment relationship.

### Work-Related Smartphone Use During Work and Work Engagement

This study demonstrated that workplace telepressure not only promotes work-related smartphone use after work but also smartphone use during work hours. We were particularly interested to study the possible effects of intensive smartphone use at work and employee work engagement. Positive psychology researchers regard work engagement as not only vital for employees, but also for organizations because it reflects a positive affective-motivational state of work-related well-being (Bakker et al., 2008; Gruman and Saks, 2011). Engaged employees are dedicated and energetic during work and are intrinsically motivated to perform as well as they can. We reasoned that work-related smartphone use during work would not necessarily relate to work engagement, but that this relationship would depend on workplace telepressure. In particular, building on the Job Demands-Resources framework, we argued that employees could see their smartphones as a demand or a resource (Bakker and Demerouti, 2017; Sonnentag, 2017). Experienced telepressure may elicit experiences of work demands rather than resources. As expected, our study showed that daily smartphone use during work was unrelated to work engagement. Moreover, we found that employees who reported high workplace telepressure experienced less work engagement on days that they used their smartphone more intensively during work. Future research could confirm this preliminary finding and test whether employees under high workplace telepressure perceive their smartphone as a burden and associate it with increased workload and longer working hours (Peters et al., 2009; Demerouti et al., 2014).

Contrary to our expectations, the combination of low workplace telepressure and work-related smartphone use was not related to increased work engagement. Employees did not seem to experience their smartphone as a job resource and may not have associated it with flexibility and increased autonomy over work tasks (Day et al., 2010). Note that employees in our sample did not benefit from using a smartphone intensively for work-related purposes during work hours. Work engagement was rather high in our sample (*M* = 4.73, *SD* = 0.79 on a scale ranging from 0 to 6), which might explain the absence of a smartphone-engagement relationship when workplace telepressure was low. As employees already experienced rather high work engagement, low workplace telepressure in combination with work-related smartphone could not further foster work engagement. Future studies could attempt to collect a more heterogenous sample with regard to work engagement and further explore the possibility that employees experiencing low telepressure may view their smartphone as a resource.

### Limitations and Future Directions

One of the strengths of the current study is that next to the relatively new concept of workplace telepressure, relevant control variables were included. This contributed to knowledge building around the telepressure concept by showing that telepressure explained unique variance in smartphone use over two domains (work and home) above and beyond workload and segmentation preference. Additionally, we learned that where telepressure is detrimental for work engagement during the day, it does not seem to affect psychological detachment in the evening. It is interesting to realize that not the psychological concept of telepressure is related to—lack of—psychological detachment, but only the behavior—work-related smartphone use—itself. A final strength is that both domains (work and home) are represented in one study, which illustrated the differential impact of telepressure across domains. Next to its strengths, the present study has some limitations that should be acknowledged and addressed in future research. One possible limitation of this study is the use of self-reports to assess the constructs. Solely using self-report measures could have led to common-method bias and social desirability biases, possibly inflating actual effects. However, most of the measured variables (e.g., workplace telepressure, work engagement) concern introspective insights that are by definition perceptual and are therefore best measured with self-report questionnaires. Thus, common-method bias should not have been a large issue in the present study (see Spector, 2006; Chan, 2009). Future studies could, however, aim to assess work-related smartphone use in a more objective manner to provide a more valid estimate of work-related smartphone use and its content. This could be achieved by designing and employing an automated smartphone app that registers all smartphone behaviors during the study period. In addition, instead of employing one questionnaire per day, multiple measurement points during the workday and during off-job time could be incorporated in a daily diary design to separate measurements and thereby reduce the possibility of common-method bias.

Another limitation is the inability to determine causality. Although the study design was longitudinal, measuring within-individual relationships and thus excluding time-invariant unobserved individual differences we cannot determine directional effects as in experimental studies. Also, our findings represent synchronous effects because all questionnaires were sent at the same time in the evening. Hence, the temporal order of the variables could not be established within our design. Contrary to what we hypothesized, it could be that employees who use their smartphones more often for work-related purposes during off-job hours, but also during work, are more inclined to experience high workplace telepressure. A lagged and/or experimental design could clarify this issue and assist in shedding some light on causality. Herein, temporally separating measurements of workday and evening variables could be beneficial. Moreover, future studies may follow and compare a group of employees experiencing high workplace telepressure with a group experiencing low workplace telepressure with the aim to examine whether the high telepressure group indeed reports increased work-related smartphone use. In another study, one could attempt to increase or decrease workplace telepressure in a laboratory situation (e.g., by clearly instructing participants to respond to messages right away or after finishing a certain task) and examine whether participants deal differently with their smartphones and as a consequence respond differently to their smartphone use. These future studies should shed more light on causality between workplace telepressure, work-related smartphone use, and affective and behavioral responses.

A final limitation of this study might be the fact that almost all employees held higher education degrees. It could be that highly educated employees deal differently with workplace telepressure and engage more or less in work-related smartphone use as compared to other samples. Thus, the results of this study can only be generalized to higher educated populations and should be replicated in other, more heterogenous samples.

Despite these limitations, the findings of this study show that workplace telepressure relates to work-related smartphone use after and during work. Moreover, work-related smartphone use hampers psychological detachment regardless of workplace telepressure, whereas work-related smartphone use during work only undermines employees’ work engagement when they experience high workplace telepressure. Given the advances in technology in our modern working world, further investigations of workplace telepressure and its precise impact on smartphone use and health and performance related outcomes seem crucial to expand our understanding of the effects of technology use.

### Theoretical and Practical Implications

This study has several implications for theory and practice. Regarding its theoretical contribution, our findings extend the relatively new area of research on workplace telepressure and work-related smartphone use during work by showing that these concepts are closely related. Adaptation to technologies may be enforced by the social environment. The omnipresence of technological devices and their frequent use by others in the work environment may set the expectation that each individual employee should frequently use these devices as well (MacCormick et al., 2012). In this way, employees may feel that they are being lived rather than having autonomy over their work, which in turn may harm their well-being, and work pleasure and engagement (e.g., Thompson and Prottas, 2006). Future studies should more often include work-related smartphone use both during and after work and test their simultaneous effects. Furthermore, future studies need to account for workplace telepressure when examining these effects. In addition, this study extends the Job Demands-Resources model by framing high workplace telepressure and subsequent intensive smartphone use as job demands and low workplace telepressure combined with intensive smartphone use as a resource. Even though smartphone use as such but also the interaction of low workplace telepressure and intensive smartphone use were unrelated to work engagement in our study, this avenue of research needs to be continued and our findings should be replicated in other samples before drawing stronger conclusions about smartphone use as a possible resource in the Job Demands-Resources model.

Further, our findings show that telepressure explains unique variance in smartphone use both during and after work after controlling for the segmentation preference of employees. On the general level, segmentation preference and telepressure are positively related, which implies that employees who prefer to have a rather impermeable boundary between work and home domains experience higher telepressure. However, apparently employees are quite successful in segmenting since segmentation preference is positively related to daily psychological detachment (*r* = 0.27, *p* < 0.01). Although we did not include the segmentation norm in this study, please note that this finding is comparable to earlier findings on the relation between segmentation norm and daily psychological detachment (*r* = 0.26, *p* < 0.01; Derks et al., 2014).

Next to these theoretical contributions, this study has also several implications for practice. It is important for employers to acknowledge that high workplace telepressure can be unfavorable for employees as the urge to respond quickly to work-related messages is highly related to work-related smartphone use after work. This may ultimately hamper psychological detachment from work. Thus, we advise that employers communicate clearly about expectations regarding responding to work-related messages and encourage employees to set clear boundaries (Barber and Jenkins, 2014; Mellner, 2016). Employers could set a good example by not contacting their employees during the evenings or at the least clearly communicate that a response is not necessary until the next workday. Generally, it is important that organizations communicate openly about organizational norms regarding working after work hours. Overtime work hours should only be voluntary, employees should not feel pressured to work during their free time (Beckers et al., 2008).

Employees can also take action themselves if they notice that they suffer from high workplace telepressure and their intensive smartphone use. They could try to resist this pressure and refrain from using their smartphone during the evenings (for work) as to experience more psychological detachment and better recovery from work.

## Conclusion

This diary study aimed to explore relevant correlates of work-related smartphone use after and during work. Our findings suggest that workplace telepressure is strongly associated with work-related smartphone use after and during work hours. Moreover, intensive smartphone use after work hampers psychological detachment regardless of experienced workplace telepressure, whereas work-related smartphone use during work can undermine work engagement, but only when there is high workplace telepressure.

## Data Availability

The raw data supporting the conclusions of this manuscript will be made available by the authors, without undue reservation, to any qualified researcher.

## Author Contributions

MVL and AvV designed the study. MVL analyzed the data with valuable input from AvV and DD. MVL wrote the first draft of the manuscript. AvV revised the first draft and added multiple sections throughout the manuscript. DD wrote sections of the discussion. All authors contributed to manuscript revision and approved the final version. The authors thank Oskar Wolthoorn for collecting the data.

## Conflict of Interest Statement

The authors declare that the research was conducted in the absence of any commercial or financial relationships that could be construed as a potential conflict of interest.
